# Supplementary material for: Effects of Two Kinds of Commercial Organic Fertilizers on Growth and Rhizosphere Soil Properties of Corn on New Reclamation Land
Source: Plants (Basel). 2022 Sep 28;11(19):2553. doi: 10.3390/plants11192553 (PMC9571666; doi:10.3390/plants11192553)
Supplement: Supplementary file 1 [file plants-11-02553-s001.zip › plants-1906055-supplementary.pdf]

Supplementary File Table S1 Effect of two kinds of commercial organic fertilizers on corn production, pH, OMC, total N, available P, available K, exchangeable Ca, exchangeable Mg

Treatments (kg/m²)	weight of air-dried Corn ears (kg/plot)			pH			OMC			Total N			available P			available K			exchangeable Ca			exchangeable Mg		
	2019	2020	2021	2019	2020	2021	2019	2020	2021	2019	2020	2021	2019	2020	2021	2019	2020	2021	2019	2020	2021	2019	2020	2021
PM-COF (0.90)	*	*	*	ns	ns	ns	*	*	*	ns	ns	*	*	*	*	*	*	*	*	ns	ns	ns	ns	ns
PM-COF (1.35)	*	*	*	ns	ns	ns	*	*	*	ns	ns	*	*	*	*	*	*	*	*	ns	ns	ns	ns	ns
PM-COF (1.80)	*	*	*	ns	ns	ns	*	*	*	ns	ns	*	*	*	*	*	*	*	*	ns	*↓	ns	ns	ns
SM-COF (0.75)	*	*	*	ns	ns	ns	*	*	*	ns	ns	*	*	*	*	*	*	*	ns	ns	ns	ns	ns	ns
SM-COF (1.05)	*	*	*	ns	ns	ns	*	*	*	ns	ns	*	*	*	*	*	*	*	ns	ns	ns	ns	ns	ns
SM-COF (1.35)	*	*	*	ns	ns	ns	*	*	*	ns	ns	*	*	*	*	*	*	*	*	ns	ns	ns	ns	ns
CCF (0.075)	*	ns	*	ns	ns	ns	ns	ns	ns	ns	ns	ns	*	*	*	*	*	*	ns	ns	ns	ns	ns	ns
Control	-	-	-	-	-	-	-	-	-	-	-	-	-	-	-	-	-	-	-	-	-	-	-	-

PMMR–OF: pig manure and mushroom residue organic fertilizer; SM–OF: sheep manure organic fertilizer; CCF: chemical compound fertilizer. ns: not significant; ↓: reduction; *: significant differences ($p < 0.05$).
